# Revisiting extraprostatic extension based on invasion depth and number for new algorithm for substaging of pT3a prostate cancer

**DOI:** 10.1038/s41598-021-93340-3

**Published:** 2021-07-06

**Authors:** Cheol Keun Park, Yeon Seung Chung, Young Deuk Choi, Won Sik Ham, Won Sik Jang, Nam Hoon Cho

**Affiliations:** 1 Department of Pathology, Severance Hospital, Yonsei University College of Medicine, 50-1 Yonsei-ro, Seodaemun-gu, Seoul, 03722 Republic of Korea; 2 Department of Urology, Severance Hospital, Yonsei University College of Medicine, Seoul, Republic of Korea

**Keywords:** Prostate cancer, Prognostic markers

## Abstract

Extraprostatic extension (EPE) is a factor in determining pT3a stage in prostate cancer. However, the only distinction in EPE is whether it is focal or non-focal, causing diagnostic and prognostic ambiguity. We substaged pT3a malignancies using classification of EPE to improve personalized prognostication. We evaluated 465 radical prostatectomy specimens with a digital image analyzer by measuring the number, radial distance and two-dimensional square area of the EPE. The most significant cut-off value was proposed as an algorithm for the pT3a substaging system to predict biochemical recurrence (BCR). A combination of the radial distance and the number of EPEs predicted BCR the most effectively. The optimal cut-off criteria were 0.75 mm and 2 mm in radial distance and multifocal EPE (hazard ratio: 2.526, C-index 0.656). The pT3a was subdivided into pT3a1, < 0.75 mm and any number of EPEs; pT3a2, 0.75–2 mm and one EPE; and pT3a3, > 2 mm and any number of EPEs or 0.75–2 mm and ≥ 2 EPEs. This combined tier was highly significant in the prediction of BCR-free survival. The combination of radial distance and number of EPEs could be used to subdivide pT3a prostate cancer and may aid in the prediction of BCR.

## Introduction

Prostate cancer is the most commonly diagnosed malignancy after lung cancer in men worldwide, and its incidence is gradually increasing^1^. In addition, it accounts for one third of mortality in Asia and Europe^2^. Radical prostatectomy (RP) is the most widely accepted curative therapeutic option for prostate cancer patients.

Pathological staging and resection margin status are intimately associated with extraprostatic extension (EPE) or whether the tumor boundaries are limited to the organ-confined zone or extend beyond the capsule. EPE is defined as cancer extension beyond the confines of prostate gland and is currently indicative of the pT3a stage^3^. The pT3a stage is an unfavorable prognostic factor that indicates cancer progression and poor patient survival outcomes^4–8^.

Although the concept of pT3a was first proposed by Epstein et al. in 1993^4,5^, some articles have defined adequate cut-off criteria^6–15^. In the initial large-scale analysis by Epstein et al., a radial EPE distance of 0.5 mm was proposed as a reasonable cut-off criterion^4^. This cut-off was lengthened to 0.75 mm in a large series of EPE algorithms by Cheng et al. and was identified as the most important parameter for prostate-specific antigen (PSA) recurrence^9^. Despite slight differences in cut-off values, most studies suggest that EPE is a significant variable that indicates poor prognosis in prostate cancer patients. However, several complicating factors must be considered.

First, the definition of an EPE is still vague and causes interobserver discrepancy in equivocal cases, even among urologic pathologists^16,17^. Additionally, several clinical variables can complicate the determination of the presence or the extent of an EPE, including the surgical procedure, the pericapsular environment and the absence of guidelines for area lacking a true capsule.

EPE might be measured more objectively via digitalized criteria than descriptive criteria. Two-dimensional (2D) areas measured using radial distance and circumferential length or the number of EPEs can be also clearly determined. In this study, we aimed to establish a new subdivision of pT3a based on a reassessment of single and/or combined factors, including a dimensional value of EPE and the number of EPEs.

## Results

### Demographic findings

After the assessment of entire cases, 1,402 tumors were confined to the prostate proper (pT2: 73.7%) and 501 tumors extended beyond the prostate capsule (pT3: 26.3%). Of the 501 RP cases, 465 cases with no evidence of seminal vesicle invasion were assigned to pT3a. Thirty-six cases showed seminal vesicle invasion and were assigned to pT3b. During the follow-up period, which lasted a minimum of 2 years, BCR was observed in 610 out of 1,903 patients (32.1%). Among the entire cases, the overall mortality and cancer-specific mortality rates were 2.3% and 1.4%, respectively. Demographic findings according to biochemical recurrence (BCR) are presented in Table 1.

**Table 1:** Demographic results of 465 EPE positive pT3a cases according to BCR.

Category	Variable	No. of cases (n = 465)	No BCR (%) (n = 210)	BCR (%) (n = 255)	*P* value
Radial distance of index EPE (mm)			1.22 ± 1.34	2.41 ± 2.10	< 0.001
Width of index EPE (mm)			5.47 ± 7.14	9.95 ± 10.9	< 0.001
Number of EPE	1	416	202 (96.2)	214 (83.9)	0.001
	2	42	7 (3.3)	35 (13.7)	
	≥ 3	7	1 (0.5)	6 (2.4)	
2D square area of index EPE (mm^2^)			12.95 ± 55.69	36.49 ± 78.07	< 0.001
Age			65.99 ± 6.99	66.10 ± 7.35	0.855
Initial PSA (ng/mL)			14.75 ± 19.40	25.18 ± 41.54	< 0.001
PGG	1	8	7 (3.3)	1 (0.39)	< 0.001
	2	126	88 (41.9)	38 (14.9)	
	3	81	46 (21.9)	35 (13.7)	
	4	71	29 (13.8)	42 (16.5)	
	5	179	40 (19.0)	139 (54.5)	
Tumor volume (cc)	≤ 2	76	58 (27.6)	18 (7.1)	< 0.001
	2–5	223	119 (56.7)	104 (40.8)	
	> 5	165	33 (15.7)	133 (52.1)	
PNI	Absent	23	15 (7.1)	8 (3.1)	0.047
	Present	442	195 (92.9)	247 (96.9)	
LVI	Absent	369	189 (90.0)	180 (70.6)	< 0.001
	Present	96	21 (10.0)	75 (29.4)	
High grade PIN	Absent	273	107 (51.0)	166 (65.1)	< 0.001
	Present	192	103 (49.0)	89 (34.9)	
AM extension	Absent	360	188 (89.5)	172 (67.5)	< 0.001
	Present	105	22 (10.5)	83 (32.5)	
BM extension	Absent	304	174 (82.9)	130 (51.0)	< 0.001
	Present	161	36 (17.1)	125 (49.0)	
CM extension	Absent	238	132 (62.9)	106 (41.6)	< 0.001
	Present	227	78 (37.1)	149 (58.4)	
VM extension	Absent	446	207 (98.6)	239 (93.7)	0.008
	Present	19	3 (1.4)	16 (6.3)	
LNM*	Absent	180	65 (90.3)	115 (70.1)	0.001
	Present	56	7 (9.7)	49 (29.9)	

BCR, biochemical recurrence; EPE, extraprostatic extension; PSA, prostate specific antigen; PGG, prostate grade group; PNI, perineural invasion; LVI, lymphovascular invasion; AM, apex margin; BM, basal margin; CM, circumferential margin; VM, vas deferens margin; LNM, lymph node metastasis.

*Evaluated in 236 cases.

### Prostate grading group (PGG) and Gleason pattern (GP) in EPE

All 1,903 RP cases were assigned to five subgroups according to PGG classification^18^: 357 cases of PGG 1, 670 cases of PGG 2, 313 cases of PGG 3, 224 cases of PGG 4 and 339 cases of PGG 5. When focusing on 501 pT3 cases, these cases were assigned to each group as follows: eight cases of PGG 1, 126 cases of PGG 2, 81 cases of PGG 3, 71 cases of PGG 4 and 179 cases of PGG 5.

**Figure 1:**
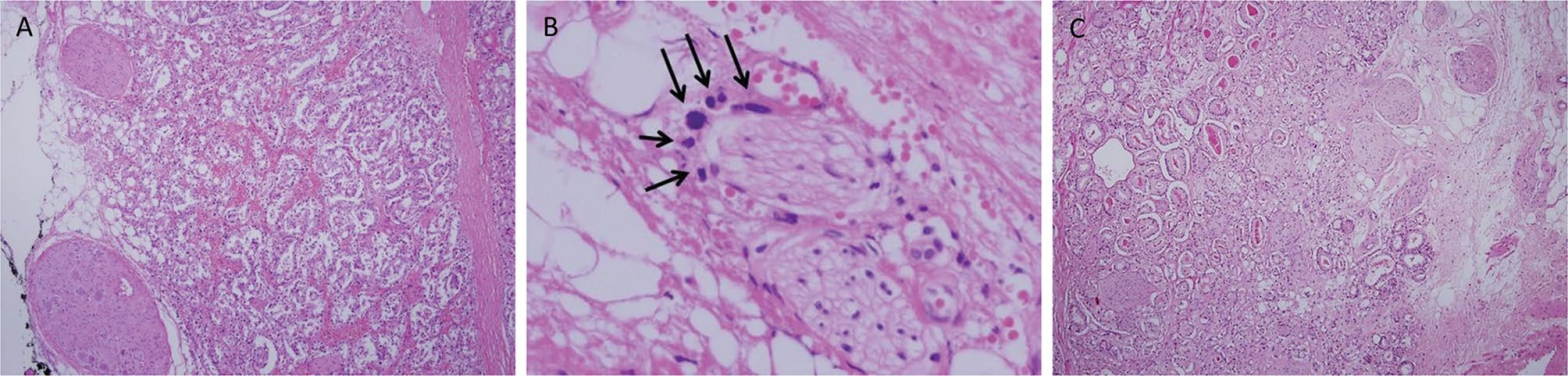
Gleason grade and extraprostatic extension (EPE). (**A**) Gleason patterns in the areas of EPE paralleled the dominant tumor pattern, usually Gleason patterns 4 and 5. (**B**) A few cancer cells scattered around the nerve outside of the capsule were often encountered and can indicate Gleason pattern 5. (**C**) In a few cases of PGG2 and 3, EPEs were scored as Gleason Grade 3, whether alone or more commonly mixed with Grade 4.

In the majority of cases, GP in the areas of EPE paralleled that of the dominant tumor nodule, usually GP 4 and 5 (Fig. 1A,B). However, in a few cases of PGG 2 and 3, GP 3 tumor was identified in the area of EPE, whether alone or more commonly mixed with GP 4 (Fig. 1C). Seminal vesicle invasion was not found in PGG 1 and was observed in six cases of PGG 2 and eight cases of PGG 3.

### Analysis of EPE in pT3a prostate cancer

All 465 pT3a cases showed one to nine EPE foci. The majority of pT3a cases showed a single focus of EPE and 49 cases (10.5%) showed multifocal EPE with two or more foci. The majority of multifocal EPEs showed two separate extensions (42 cases), and seven cases showed three to nine EPE foci.

For radial distance, 99 cases (21.3%) extended beyond the capsule less than 0.75 mm, 283 cases (60.9%) extended between 0.75 and 2 mm, and 83 cases (17.8%) extended more than 2 mm across the capsule.

### Cut-off value of radial distance and 2D square area

To determine the optimal cut-off value of radial distance and 2D square area for the prediction of BCR, Cox regression was performed with various cut-off values. For radial distance, the cut-off value of 0.75 mm showed a significant hazard ratio (HR) with the highest C-index. For 2D square area, the cut-off value of 2.00 mm^2^ showed the highest C-index. However, HR was not significant when comparing cases with 2D square area ≤ 0.50 mm^2^ to those with 2D square area 2.00–5.00 mm^2^. After adjusted with various clinicopathologic factors, the cut-off value of 0.75 mm showed significant HR except the comparison between ≤ 0.75 mm and > 5.00 mm (Supplementary Table S1).

### Prognostic impact of EPE parameters on pT3a prostate cancer patients’ survival

We investigated which EPE parameter is associated with patient survival. On Cox regression, more than one EPE foci (HR 1.534), radial distance > 0.75 mm (HR 1.219) and 2D square area > 2.00 mm^2^ (HR 1.004) were all significantly associated with BCR (*P* < 0.0001 for all). In cancer-specific survival, only radial distance > 0.75 mm showed a significant correlation (HR 1.258, *P* = 0.016). In addition, it showed the highest C-index for BCR (0.648) and cancer-specific survival (0.724) compared to more than one EPE foci and 2D square area > 2.00 mm^2^. After adjusted with various clinicopathologic factors, only radial distance > 0.75 mm showed a significant correlation in BCR (HR 2.228, *P* = 0.014). (Table 2).

**Table 2:** Cox regression analysis of EPE parameters to affect pT3a prostate cancer patients’ survival.

Category	Variable	Unadjusted		Adjusted*		C-index
		HR (95% CI)	*P*-value	HR (95% CI)	*P*-value	
BCR	Radial distance ≤ 0.75 mm	1		1		
	Radial distance > 0.75 mm	1.219 (1.166–1.274)	< 0.001	2.228 (1.175–4.226)	0.014	0.648
	2D square area ≤ 2.00 mm^2^	1		1		
	2D square area > 2.00 mm^2^	1.004 (1.002–1.005)	< 0.001	1.525 (0.937–2.484)	0.090	0.641
	Number of EPEs ≤ 1	1		1		
	Number of EPEs > 1	1.534 (1.377–1.710)	< 0.001	1.355 (0.933–1.968)	0.110	0.612
Overall survival	Radial distance ≤ 0.75 mm	1		1		
	Radial distance > 0.75 mm	1.146 (0.967–1.359)	0.117	0.909 (0.333–2.484)	0.853	0.602
	2D square area ≤ 2.00 mm^2^	1		1		
	2D square area > 2.00 mm^2^	1.003 (0.998–1.007)	0.288	0.865 (0.349–2.145)	0.754	0.607
	Number of EPEs ≤ 1	1		1		
	Number of EPEs > 1	1.345 (0.960–1.884)	0.085	1.451 (0.608–3.462)	0.402	0.604
Cancer specific survival	Radial distance ≤ 0.75 mm	1		1		
	Radial distance > 0.75 mm	1.258 (1.044–1.514)	0.016	2.880 (0.368–22.156)	0.313	0.724
	2D square area ≤ 2.00 mm^2^	1		1		
	2D square area > 2.00 mm^2^	1.004 (0.998–1.010)	0.191	1.975 (0.426–9.153)	0.385	0.694
	Number of EPEs ≤ 1	1		1		
	Number of EPEs > 1	1.393 (0.886–2.191)	0.152	2.463 (0.744–8.152)	0.140	0.665

*Adjusted for age, initial PSA level, prostate grade group, tumor volume, perineural invasion, lymphovascular invasion, apex margin extension, basal margin extension, circumferential margin extension, vas deferens margin extension, lymph node metastasis.

### Subdivision of pT3a prostate cancer and its prognostic impact

After identifying both radial distance > 0.75 mm and more than one EPE foci as candidates to predict short BCR-free survival, we surveyed these factors in more detail to define new parameters for the prediction of BCR-free survival. When combining these two parameters, it predicts the BCR of pT3a patients on Cox regression analysis with a C-index of 0.656. Based on these results, we classified pT3a into three subgroups: pT3a1, ≤ 0.75 mm and any number of EPEs; pT3a2, 0.75–2 mm and one EPE; and pT3a3, > 2 mm and any number of EPEs or 0.75–2 mm and more than two EPEs (Table 3).

**Table 3:** Cox regression analysis of BCR using radial distance 0.75 mm and number of EPE combinations.

Category	n ≤ 1	n > 1			
r ≤ 0.75	(1,1)	(1, 2)			
0.75 < r ≤ 2	(2, 1)	(2, 2)			
2 < r ≤ 5	(3, 1)	(3, 2)			
r > 5	(4, 1)	(4, 2)			

Radial (1,2,3,4) Number (1,2)	HR	95% CI		*P*-value	c-index
		Lower	Upper		
Ref = (1, 1)	1				
(2, 1)	1.665	1.1984	2.312	0.0024	0.656
(3, 1)	3.657	2.3449	5.704	< 0.0001	
(1, 2)	1.753	0.8098	3.796	0.1542	
(2, 2)	2.884	2.0135	4.131	< 0.0001	
(3, 2)	4.237	2.968	6.049	< 0.0001	
(4, 1) + (4, 2)	4.203	2.6148	6.756	< 0.0001	

n: number of EPE, r: radial distance of EPE (mm).

**Figure 2:**
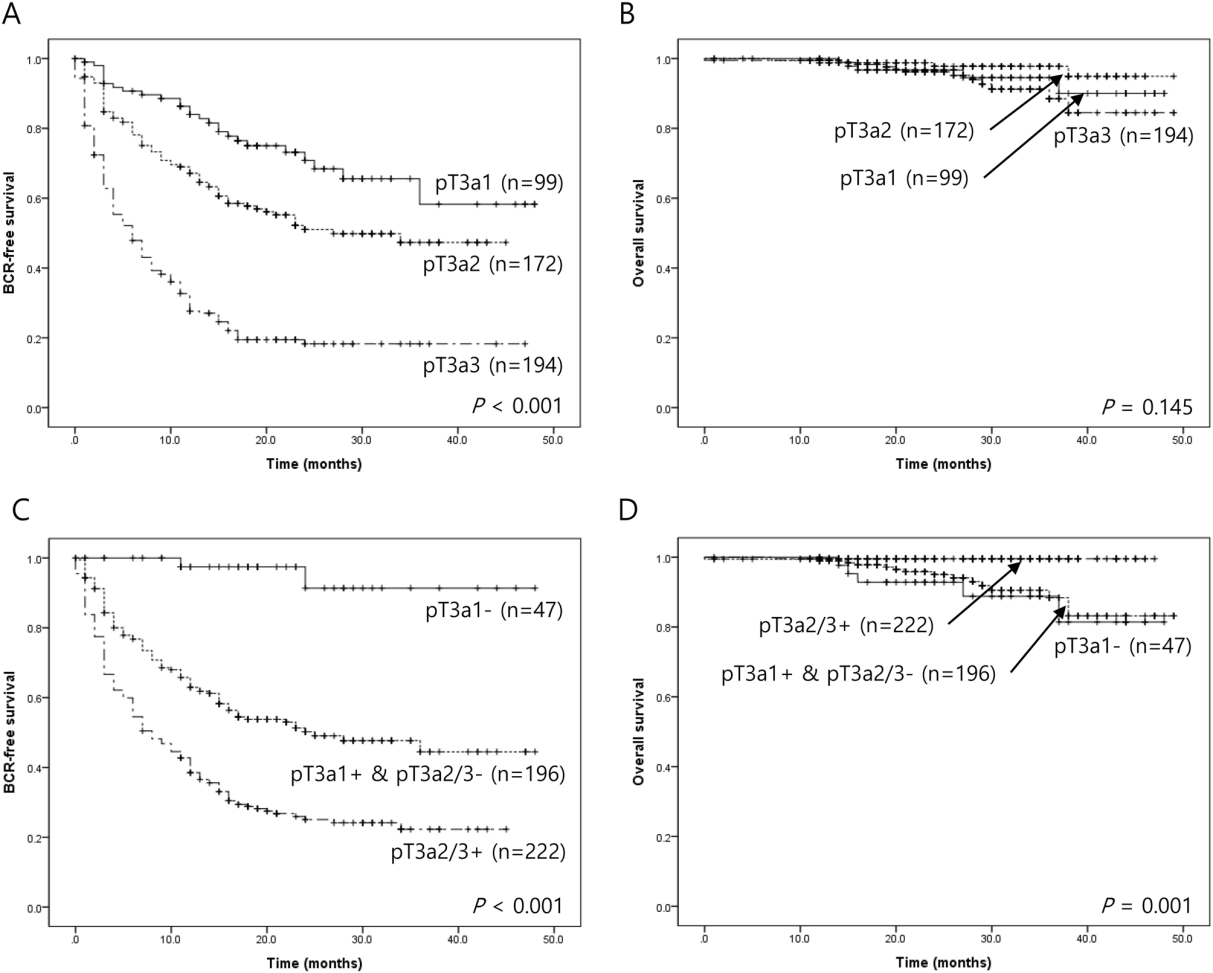
Kaplan-Meyer survival analysis according pT3a substage and the combination of pT3a substage and circumferential margin (CM) status. (**A**) The biochemical recurrence (BCR)-free survival was significantly different among pT3a subgroups (*P* < 0.001). (**B**) No significant differences were identified in OS (*P* = 0.145). (**C**) After the combination of pT3a substage and CM status, significant differences were identified among 3 subgroups (*P* < 0.001). (D) pT3a2/3 + subgroup showed longer OS than other subgroups (*P* = 0.001).

According to this new pT3a classification system, we performed Kaplan–Meier analysis. It showed significant differences among pT3a subgroups in BCR-free survival (*P* < 0.001; Fig. 2A). In univariate analysis, higher initial PSA level (*P* < 0.001), tumor volume 2-5 cc (*P* = 0.012), tumor volume > 5 cc (*P* < 0.001), presence of lymphovascular invasion (*P* < 0.001), presence of apex margin extension (*P* < 0.001), presence of basal margin extension (*P* < 0.001), presence of circumferential margin (CM) extension (*P* < 0.001), presence of vas deferens margin extension (*P* < 0.001), pT3a1 substage (*P* = 0.006), pT3a2 substage and presence of lymph node metastasis (*P* < 0.001) were associated with shorter BCR-free survival. In multivariate analysis, presence of apex margin extension (*P* = 0.014), presence of basal margin extension (*P* = 0.015), presence of circumferential margin (CM) extension (*P* = 0.039; Table 4) were associated with shorter BCR-free survival. In Kaplan–Meier analysis of overall survival (OS), no significant difference was identified (*P* = 0.145; Fig. 2B).

**Table 4:** Univariate and multivariate analysis of 465 prostate cancer cases on BCR-free survival.

Category	Variable	Univariate		Multivariate	
		HR (95% CI)	P-value	HR (95% CI)	P-value
Age (years)*	≤ 67	1		–	
	> 67	1.119 (0.875–1.431)	0.370	–	–
Initial PSA (ng/mL)**	≤ 11.75	1		1	
	> 11.75	1.663 (1.293–2.138)	< 0.001	0.954 (0.687–1.325)	0.779
Prostate grade group	1	1		–	
	2	2.677 (0.643–11.146)	0.176	–	–
	3	4.714 (1.136–19.570)	0.033	–	–
	4	6.068 (1.469–25.066)	0.013	–	–
	5	10.162 (2.512–41.113)	0.001	–	–
Tumor volume (cc)	≤ 2	1		1	
	2–5	1.928 (1.154–3.221)	0.012	1.438 (0.629–3.286)	0.389
	> 5	4.832 (2.912–8.017)	< 0.001	1.448 (0.605–3.463)	0.406
PNI	Absent	1		–	
	Present	0.995 (0.511–1.935)	0.987	–	–
LVI	Absent	1		1	
	Present	2.317 (1.766–3.040)	< 0.001	1.299 (0.901–1.872)	0.161
AM extension	Absent	1		1	
	Present	2.110 (1.622–2.744)	< 0.001	1.538 (1.090–2.170)	0.014
BM extension	Absent	1		1	
	Present	2.645 (2.065–3.389)	< 0.001	1.604 (1.096–2.348)	0.015
CM extension	Absent	1		1	
	Present	1.870 (1.454–2.405)	< 0.001	1.487 (1.020–2.168)	0.039
VM extension	Absent	1		1	
	Present	2.798 (1.926–4.064)	< 0.001	1.194 (0.728–1.958)	0.483
pT3 substage	pT3a1	1		1	
	pT3a2	1.854 (1.196–2.871)	0.006	1.676 (0.847–3.316)	0.138
	pT3a3	5.067 (3.353–7.656)	< 0.001	2.640 (1.366–5.104)	0.004
LNM***	Absent	1		1	
	Present	2.367 (1.686–3.323)	< 0.001	1.275 (0.861–1.887)	0.225

BCR, biochemical recurrence; PSA, prostate specific antigen; PGG, prostate grade group; PNI, perineural invasion; LVI, lymphovascular invasion; AM, apex margin; BM, basal margin; CM, circumferential margin; VM, vas deferens margin; LNM, lymph node metastasis.

*Median age of 465 patients was 67.0 years.

**Median level of initial PSA was 11.75 ng/mL.

***Evaluated in 236 cases.

### EPE according to CM status

For the impact of EPE and CM status on BCR-free survival, a comparison of hazard ratio between a pT2-CM positive group and pT3a-CM negative group was made. When pT3a substaging overrides CM status to predict BCR, pT3a1 and the negative CM group (pT3a1-) showed significantly poor survival (*P* = 0.0426) in BCR compared to pT2 and the positive CM group (pT2 + ; Supplementary Table S2).

Considering EPE and CM status, pT3a2 and pT3a3 were not significantly different. When pT3a2 and pT3a3 are combined, pT3a1 and positive CM (pT3a1 +) showed no significant difference with pT3a2/3 and negative CM (pT3a2/3-; *P* = 0.6808). Both groups demonstrated similar HR (5.053, and 5.204, respectively) compared with the pT2- group. After combining pT3a1 + and pT3a2/3- subgroup, BCR-free survival OS were significantly different among subgroups (*P* < 0.001 and 0.001, respectively; Fig. 2C,D).

## Discussion

**Figure 3:**
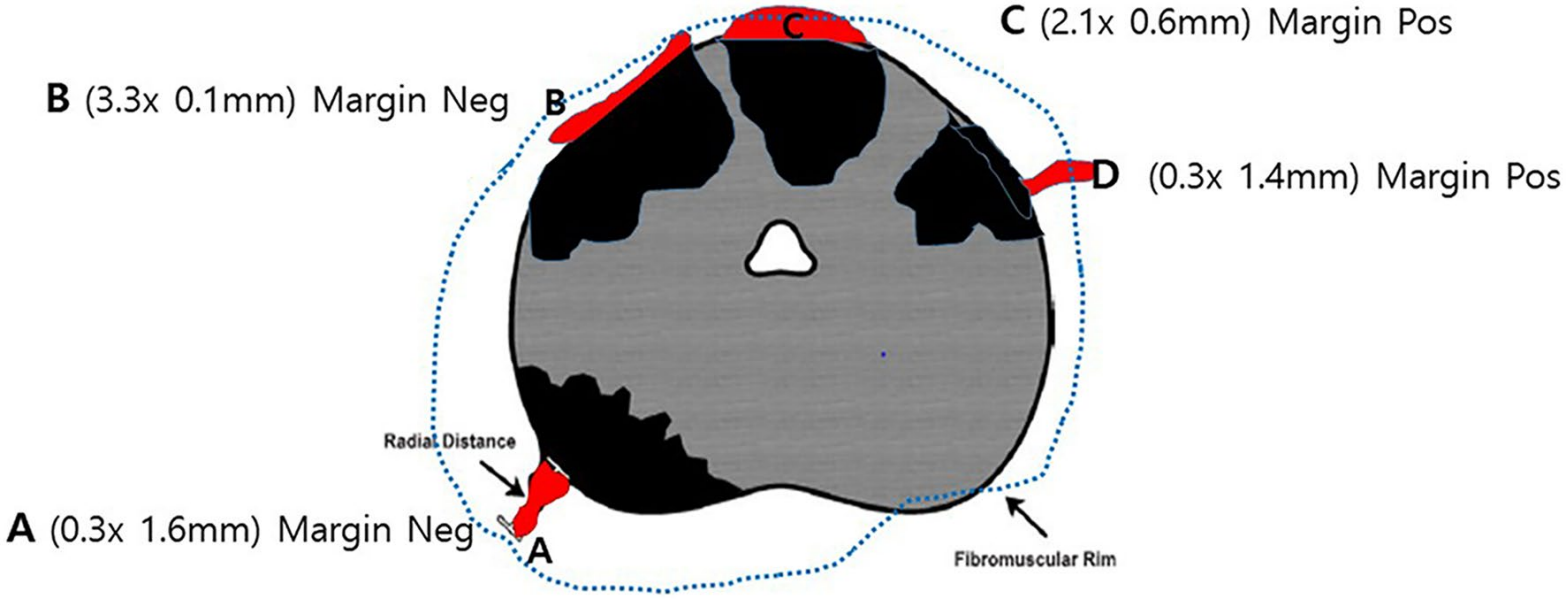
Schematic diagram of various cases of EPE. In general, EPE erupts from the origin of the tumor nodule. In cases of multifocal tumor nodules, which are frequently observed, multiple EPE foci can be present. When only radial distance is measured, the “A” type could be an EPE index because it is the longest in radial distance. However, in comparison with 2D areas that include circumferential length, the “C” type can be an EPE index. Another dilemma is whether EPE is associated with circumferential margin involvement. The combination of “C” type in 2D or “D” type in radial distance could be a more useful EPE index.

The presence of EPE is an unfavorable prognostic factor that indicates poor survival outcomes^4–8^. However, the assessment of EPE varies depending on the evaluation method, especially in cases of multiple EPE foci (Fig. 3). In addition, resection margin status may influence tumor staging despite actually similar extents of EPE. Therefore, we evaluated EPE using various measurement criteria and stratified patient prognosis according to the EPE assessment results, especially in pT3a patients.

EPE was primarily composed of PGG 4 or 5, however, Gleason pattern 3 was the main component of EPE in a few cases of PGG 2 and 3. In addition, eight cases (1.7% of pT3a cases) of PGG 1 with larger than 2 cc of intraglandular tumor volume showed EPE. A recent study reported that the incidence of PGG 1 was 0.28% in pT3a and no incidence in pT3b, which is in accordance with our results^19^. Our findings suggest that PGG 1 could be pT3a in the case of a large volume. In addition, Gleason pattern 3 in PGG 2 or 3 can be the main component of EPE, suggesting that the worst grade is not always a determining factor for poor prognosis.

Many predictive values of EPE related to BCR have been investigated^4–15^. Focal EPEs contain a few neoplastic glands exterior to the prostate, and established EPEs show more extensive extraprostatic spread^4,5^. Several attempts have been made to optimize these definitions and documented optimal cut-off radial distances at 0.75 mm^9^, 0.6 mm (high power restriction)^6^, or 1.1 mm (low power restriction)^12^. Recently, one study observed that EPEs > 1 mm and a length of surgical margin positivity > 2 mm was an independent predictor of BCR (AUC 0.713)^15^. In this study, we revealed that radial distance of EPE is significantly associated with EPE and showed excellent interobserver agreement (interclass correlation coefficient = 0.999, *P* < 0.001). In addition, pT3a stage can be subclassified by combining radial distance with cut off value of 0.75 mm and number of EPE.

In the 2009 International Society of Urological Pathology consensus conference, a consensus on the stratification of EPE to focal and established was reached; however, a specific method for the evaluation of EPE did not obtain a consensus among the delegates^3^. For better reproducibility and clinical correlation, we propose some additions: (1) The confirmation of multifocal EPEs should be done with care. Overlapping tumors in serial sections can show irregular boundaries in multiple foci, making it difficult to determine if they are separated or continuously adjoined. (2) Multifocal EPEs require an EPE index, which should be defined as the longest radial distance.

Most studies that suggested cut-off values used the original EPE definition, focal versus non-focal, measured by light microscopy. Although the majority of studies measured EPEs with conventional light microscopy, which is practical and convenient, light microscopy is rough and less reproducible. To address this concern, we measured radial and circumferential lengths by digital image scanning with an image analyzer. The power of the digital microscopy in EPE measurement was shown to be similar to but more accurate than conventional microscopy in a previous study^20^.

A recent study analyzed radial and circumferential length individually and found that both factors were significantly associated with BCR^15^. There have been, however, no investigations into the 2D square area of EPE. Using the concept of 2D square area of EPE, these extensions could be classified as non-focal (established) extensions. However, whether EPE extent includes circumferential length for prognostication remains to be determined. In addition, we found that the 2D square area was significantly associated with BCR, but to a lesser extent than the radial distance. The apparent disadvantage of the 2D square area of EPE is the difficulty in calculation compared to the radial distance alone.

Some studies have reported that the extension to resection margin is associated with BCR^21,22^. However, the prognostic significance of the tumor extent in positive resection margin is still controversial^8,23–25^. Recently, a few studies emphasized the prognostic significance of EPE and multifocality of positive resection margin on BCR^10,15^. However, the investigation of prognostic impact on BCR with a combination of EPE and CM status have not been performed yet. Our study showed that pT3a substaging system considering CM status also proportionately increased the hazard ratio up to 7.61-fold. However, pT3a2 was not significant in the hazard ratio of pT3a3 in the negative CM status.

Defining multifocal EPEs remains challenging. One study defined multiple EPEs as more than one EPE focus < 0.8 mm in radial distance. This study also demonstrated that the presence of multiple EPEs defined in this way was a significant independent factor of BCR together with established EPE^26^. In the present study, we defined multifocality as cases of > 1 EPE foci, regardless of the distance. As a result, the number of EPEs was very significant, even without considering the radial distance of each EPE. Moreover, the EPE number itself was as significantly associated with BCR as the radial distance, and the combined module significantly predicted BCR.

The evaluation of EPE via digital microscopy has some advantages. First, this method can provide the precise measurement of EPE and high reproducibility. In addition, it may useful for the improvement of radiological-pathological correlation and validation of prostate cancer magnetic resonance imaging using machine learning application^27,28^.

On the contrary, this method also has some disadvantages and limitations. First, we used a digital microscopy device for the precise measurement of EPE area. However, it may be the hurdle for hospitals or clinics without this digital device. Therefore, further study regarding measurement method, digital microscopy versus light microscopy with naked eyeball is necessary. Secondly, the effect of bladder neck invasion has not been evaluated because the majority of cases underwent the surgical procedure that spares the bladder neck. Since the bladder neck invasion is considered as pT3a, some cases classified as pT2 may have bladder neck invasion and their prognosis may be underestimated. Further studies are necessary for the prognostication of cases with bladder neck invasion.

In addition, our study was performed on the single cohort from the single institute. Thus, further study is necessary to validate our new subclassification system for pT3a prostate cancer and compare with the current dichotomous classification for EPE in other cohorts composed of multi-center and different ethnic groups.

In conclusion, we propose a three-tiered pT3a subdivision system for the prediction of BCR, based on the number and radial distance of EPE using digital microscopy. By considering the number of EPEs, our proposed three-tiered subdivision system may show more significant prognostic discrimination.

## Materials and methods

### Patients and samples

After excluding patients with prior hormonal therapy, a total of 1,903 consecutive RP specimens obtained from Severance Hospital from 2014 to 2017 were included in this study.

The Institutional Review Board of Severance Hospital approved this study and the informed consent was waived (4–2020-0622). The study adhered to the principles of the declaration of Helsinki. All cases were periodically followed and checked for metastasis by imaging and PSA evaluation. The first postoperative PSA was obtained 6–8 weeks after the operation. BCR was defined as a postoperative PSA equal to or more than 0.1 ng/mL.

All selected RP cases were re-evaluated by two independent pathologists (CKP and YSC) to assess total GS and presence of EPE. The confirmation of entire cases was performed by the urologic pathologist (NHC). For cases with EPE, assignment of a GS to the EPE area was performed, according to a previous report^29^. Patients were further stratified into groups based on prognostic variables including pathologic features of the cancer, GS, PGG^18^, seminal vesicle or vas deferens invasion, intraglandular tumor volume, surgical margin status, lymph node status, and EPE status.

### Measurement of EPE

Thirty-six cases showing seminal vesicle invasion were excluded. Finally, 465 cases were selected for the analysis of EPE. Two major types of EPE contour were defined: broad-based sessile extensions and church-spire shaped-protrusions. When the radial distance of EPE is more than 2 times of circumferential length, it is defined as church-spire type. The other cases were defined as broad-based sessile extension. All EPEs were measured in radial distance and 2D square area using digital microscopy (Cellsens Standard 2.1, Olympus, Tokyo, Japan) by two independent pathologists (CKP and NHC).

Radial distance is defined as the distance that the tumor protrudes perpendicularly beyond the outer margin of the capsule. The 2D square area was defined as the sum of the EPE area calculated by multiplication of radial distance and circumferential length, regardless of the shape. Circumferential length was measured at the largest width between both sides of the involved fat. When tumors protruded to the extraprostatic boundary without fat tissue, tracing to the nearest fat line was accepted as baseline. An imaginary line of the adjacent capsule was used as the baseline in zones that lacked the capsule layer (anterior and urethral zone). The example of measurement for radial distance, circumferential length and 2D square area of EPE is presented as Supplementary Figure S1.

In cases of discontinuous or multiple EPEs, the continuity with tumor nodules was considered. When two EPEs connected with the same nodule, it was considered one EPE; when EPEs arose from separate tumors, they were counted as more than one EPE. The number of EPEs in each case was recorded and cases were categorized into two groups for further evaluation: cases containing one EPE and those with > 1 EPE foci.

### Assessment of unusual EPE cases

**Figure 4:**
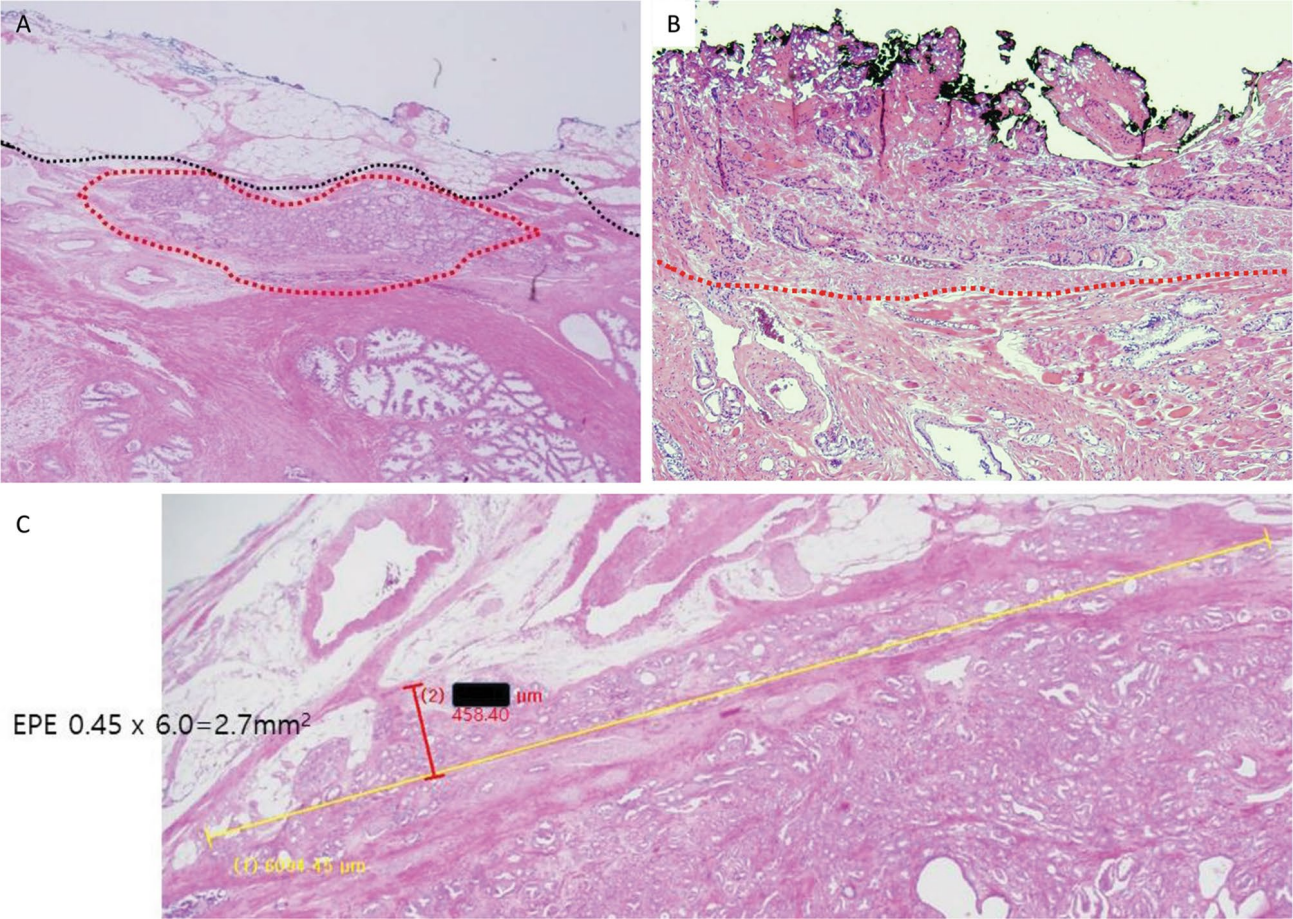
(**A**) An example of a pT2 case. The tumor showed definite protrusion beyond the prostate proper at the level of thick vasculature. However, fat pads were not apparently involved. In this case, we suggest a T2 classification. Although such cases are often encountered, they should not be dealt with as T3 cases. (**B**) The presence of tumor cells in anterior muscle bundles without neurovascular bundles causes controversy in the evaluation of EPE. Most accepted guidelines define EPEs as extending beyond the contour of the proper tissue. (**C**) EPE showed extensively broad-based sessile extensions of tumor beyond the capsule and fat pad**.**

We encountered several unusual cases where it was not possible to clearly measure the length extending beyond the prostate proper. One case showed that the tumor appeared to extend to the periprostatic soft tissue beyond the prostate proper. However, thick-walled vasculature was in the vicinity of the extending tumor and did not involve the fat pads. In this case, the tumor appeared to extend beyond the prostate capsule but did not invade into any fat pad. Therefore, it was classified as pT2 (Fig. 4A).

Five cases showed tumor cells in anterior muscle bundles without neurovascular bundles. For these cases, we defined EPEs as extending beyond the contour of the proper tissue (Fig. 4B). In addition, some cases showed EPE with extensively broad-based sessile extensions of tumor beyond the capsule and fat pad (Fig. 4C). These tumors had a short radial distance of less than 0.5 mm and wide circumferential length of more than 6 mm, which blurred the distinction between truly focal and non-focal extensions.

### Statistical analysis

Student’s *t*-test and chi-square test were used for continuous and categorical variables, respectively. BCR-free survival was determined by Kaplan–Meier and Cox proportional hazards regression analysis. Harrell’s c-index was calculated in Cox proportional hazards regression as previously described^30,31^. Statistical significance was assumed when *P* < 0.05. All statistical data were analyzed by R package, version 3.4.3 (http://www.R-project.org).

## Supplementary Information

Supplementary Information 1.

Supplementary Information 2.
