# Supplementary figures and images for: Revisiting extraprostatic extension based on invasion depth and number for new algorithm for substaging of pT3a prostate cancer
Source: Sci Rep. 2021 Jul 6;11:13952. doi: 10.1038/s41598-021-93340-3 (PMC8260727; doi:10.1038/s41598-021-93340-3)

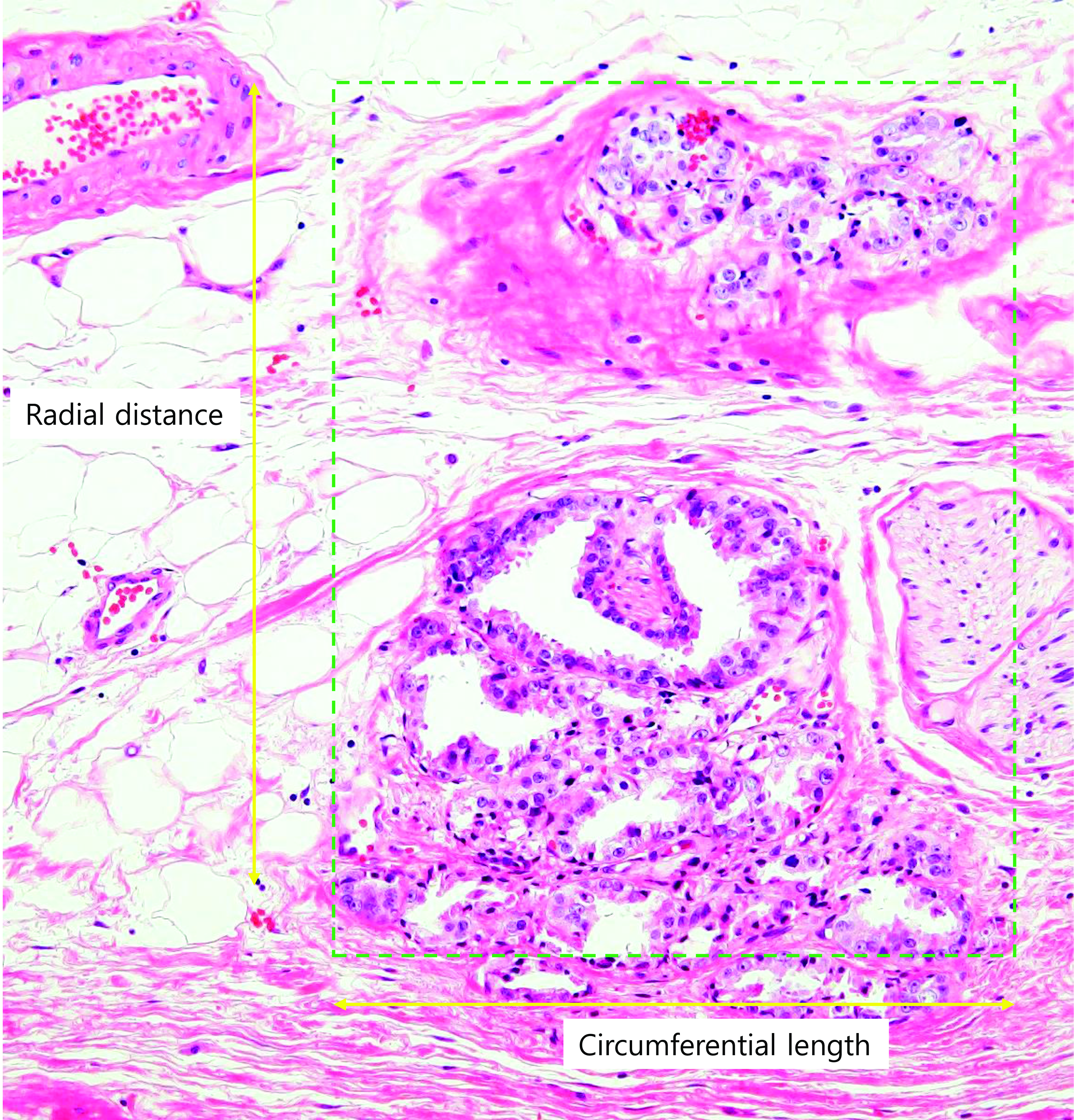

Supplement: Supplementary file 1 — Supplementary Information 1. [file 41598_2021_93340_MOESM1_ESM.tif]
