# Supplementary material for: Revisiting extraprostatic extension based on invasion depth and number for new algorithm for substaging of pT3a prostate cancer
Source: Sci Rep. 2021 Jul 6;11:13952. doi: 10.1038/s41598-021-93340-3 (PMC8260727; doi:10.1038/s41598-021-93340-3)
Supplement: Supplementary file 2 — Supplementary Information 2. [file 41598_2021_93340_MOESM2_ESM.docx]

**Original Article**

**Revisiting extraprostatic extension based on invasion depth and number for new algorithm for substaging of pT3a prostate cancer**

Cheol Keun Park, MD,^1^ Yeon Seung Chung, MD,^1^ Young Deuk Choi, MD, PhD,^2^ Won Sik Ham, MD, PhD,^2^ Won Sik Jang, MD, PhD,^2^ and Nam Hoon Cho, MD, PhD^1^

^1^Department of Pathology, Severance Hospital, Yonsei University College of Medicine, Seoul, Republic of Korea

^2^Department of Urology, Severance Hospital, Yonsei University College of Medicine, Seoul, Republic of Korea

**Supplementary Tables**

**Supplementary Table 1.** Cox regression analysis of cut-off value for radial distance and 2D square area of EPE to affect BCR

| Category | Variable | Unadjusted | | Adjusted* | | C-index |
| --- | --- | --- | --- | --- | --- | --- |
|  |  | HR (95% CI) | *P*-value | HR (95% CI) | *P*-value |  |
| Radial distance cut-off 0.50 mm | ≤ 0.50 mm | 1 |  | 1 |  | 0.611 |
|  | 0.50-1.00 mm | 1.085 (0.590-1.993) | 0.794 | 1.024 (0.431-2.435) | 0.957 |  |
|  | > 1.00 mm | 2.553 (1.962-3.321) | < 0.001 | 2.482 (1.285-4.792) | 0.007 |  |
| Radial distance cut-off 0.75 mm | ≤ 0.75 mm | 1 |  | 1 |  | 0.644 |
|  | 0.75-2.00 mm | 1.461 (1.035-2.064) | 0.032 | 2.036 (1.059-3.913) | 0.033 |  |
|  | 2.00-5.00 mm | 2.277 (1.641-3.160) | < 0.001 | 2.659 (1.330-5.318) | 0.006 |  |
|  | > 5.00 mm | 3.935 (2.911-5.319) | < 0.001 | 2.091 (0.926-4.722) | 0.076 |  |
| Radial distance cut-off 1.00 mm | ≤ 1.00 mm | 1 |  | 1 |  | 0.632 |
|  | 1.00-2.00 mm | 1.972 (1.471-2.644) | < 0.001 | 1.556 (0.984-2.460) | 0.059 |  |
|  | 2.00-5.00 mm | 3.380 (2.547-4.485) | < 0.001 | 1.854 (1.181-2.910) | 0.007 |  |
|  | > 5.00 mm | 3.500 (2.223-5.511) | < 0.001 | 1.466 (0.780-2.755) | 0.234 |  |
| 2D square area cut-off 0.50 mm^2^ | ≤ 0.50 mm^2^ | 1 |  | 1 |  |  |
|  | 0.50-1.00 mm^2^ | 2.180 (1.330-3.572) | 0.002 | 2.173 (0.644-7.339) | 0.211 | 0.578 |
|  | > 1.00 mm^2^ | 2.278 (1.700-3.054) | < 0.001 | 2.077 (0.832-5.189) | 0.118 |  |
| 2D square area cut-off 2.00 mm^2^ | ≤ 2.00 mm^2^ | 1 |  | 1 |  |  |
|  | 2.00-5.00 mm^2^ | 1.256 (0.823-1.919) | 0.291 | 0.840 (0.434-1.626) | 0.605 | 0.627 |
|  | 5.00-10.00 mm^2^ | 1.611 (1.082-2.398) | 0.019 | 1.699 (0.919-3.143) | 0.091 |  |
|  | > 10.00 mm^2^ | 3.002 (2.306-3.908) | < 0.001 | 1.862 (1.118-3.101) | 0.017 |  |
| 2D square area cut-off 10.00 mm^2^ | ≤ 10.00 mm^2^ | 1 |  | 1 |  |  |
|  | 10.00-50.00 mm^2^ | 2.301 (1.770-2.992) | < 0.001 | 1.420 (0.943-2.139) | 0.083 | 0.620 |
|  | > 50.00 mm^2^ | 3.447 (2.538-4.681) | < 0.001 | 1.555 (0.944-2.560) | 0.093 |  |
| *Adjusted for age, initial PSA level, prostate grade group, tumor volume, perineural invasion, lymphovascular invasion, apex margin extension, basal margin extension, circumferential margin extension, vas deferens margin extension, lymph node metastasis | | | | | | |

**Supplementary Table 2.** Cox regression analysis of combination of EPE and circumferential margin status.

| Variable | HR (95% CI) | *P*-value |
| --- | --- | --- |
| EPE negative & negative margin group, pT2- (group 1) | 1 |  |
| pT2 & positive margin group, pT2+ (group 2) | 2.446 (1.917-3.121) | < 0.0001 |
| pT3a1 & negative margin group, pT3a1- (group 3) | 3.298 (2.497-4.357) | < 0.0001 |
| pT3a1 & positive margin group, pT3a1+ (group 4) | 5.053 (3.871-6.595) | < 0.0001 |
| pT3a2/3 & negative margin group, pT3a2/3- (group 5) | 5.204 (4.073-6.650) | < 0.0001 |
| pT3a2/3 & positive margin group, pT3a2/3+ (group 6) | 7.261 (5.987-8.806) | < 0.0001 |

| Post hoc comparison | *P*-value | *P*-value* |
| --- | --- | --- |
| 1 , 2 | < 0.0001 | < 0.0001 |
| 1 , 3 | < 0.0001 | < 0.0001 |
| 1 , 4 | < 0.0001 | < 0.0001 |
| 1 , 5 | < 0.0001 | < 0.0001 |
| 1 , 6 | < 0.0001 | < 0.0001 |
| 2 , 3 | 0.0426 | 0.6394 |
| 2 , 4 | < 0.0001 | < 0.0001 |
| 2 , 5 | < 0.0001 | < 0.0001 |
| 2 , 6 | < 0.0001 | < 0.0001 |
| 3 , 4 | 0.0062 | 0.0931 |
| 3 , 5 | 0.0018 | 0.0272 |
| 3 , 6 | < 0.0001 | < 0.0001 |
| 4 , 5 | 0.6808 | > 0.9999 |
| 4 , 6 | 0.0017 | 0.0257 |
| 5 , 6 | 0.0034 | 0.0512 |

*Bonferroni corrected p-value

**Supplementary Figure Legend**

**Supplementary Figure 1.** The example of the measurement for radial distance, circumferential length and 2D square area of EPE.

The radial distance and circumferential length are displayed as yellow colored line. The 2D square area is the area marked with green dotted line.
